# Exploratory Diagnostic Utility of a Modified Feng-Derived Preoperative Risk Score for Predicting Occult Contralateral Carcinoma in Filipino Patients With Clinically Solitary Papillary Thyroid Carcinoma

**DOI:** 10.7759/cureus.109610

**Published:** 2026-05-25

**Authors:** Iverick Pierce U Militante, Mark Philip C Guinocor

**Affiliations:** 1 General Surgery, Chong Hua Hospital, Cebu, PHL

**Keywords:** diagnostic accuracy, filipino patients, occult contralateral carcinoma, papillary thyroid carcinoma, risk score, thyroid lobectomy, total thyroidectomy

## Abstract

Introduction: The optimal surgical extent for clinically solitary papillary thyroid carcinoma remains debated. Total thyroidectomy addresses possible occult contralateral carcinoma but increases morbidity and requires lifelong hormone replacement, while thyroid lobectomy may avoid overtreatment but risks leaving occult disease. This study evaluated a modified preoperative risk score using clinical and ultrasonographic findings to predict occult contralateral carcinoma in Filipino patients.

Materials and methods: This single-center retrospective diagnostic accuracy study included 135 adult patients who underwent total thyroidectomy for clinically solitary papillary thyroid carcinoma at Chong Hua Hospital, Cebu, from 2019 to 2023. Preoperative variables included a primary tumor measuring more than 1 cm, presence of benign-appearing additional nodules, suspected extrathyroidal extension, suspected central lymph node involvement, and suspected lateral lymph node involvement. The modified preoperative score was calculated for each patient using preoperative neck ultrasonography and physical examination findings. The primary outcome was occult contralateral carcinoma on final histopathology. Model discrimination was examined by plotting a receiver operating characteristic curve and calculating the area under the curve.

Results: Occult contralateral carcinoma was identified in 19 of 135 patients. The adapted preoperative scoring system demonstrated strong discriminative ability in this cohort, yielding an area under the receiver operating characteristic curve of 0.91 (95% CI, 0.845 to 0.976). Using a threshold of at least four points, the score showed apparent sensitivity of 94.7%, specificity of 87.1%, positive predictive value of 54.6%, negative predictive value of 99.0%, and overall accuracy of 88.2%.

Conclusions: The modified preoperative risk score showed promising apparent diagnostic performance for predicting occult contralateral carcinoma among Filipino patients with clinically solitary papillary thyroid carcinoma. Its high negative predictive value suggests potential usefulness as an adjunctive rule-out tool during preoperative counseling, but only when interpreted together with established guideline-based risk stratification, tumor characteristics, nodal assessment, patient preferences, and surgeon judgment. The score should not be used as an independent determinant of surgical extent, particularly given its modest positive predictive value. Prospective multicenter validation is needed before routine clinical use.

## Introduction

Papillary thyroid carcinoma is the most prevalent endocrine malignancy, representing over 80% of all thyroid cancers worldwide [1]. While generally associated with an excellent prognosis, the appropriate extent of initial surgery for clinically unilateral, low- to intermediate-risk papillary thyroid carcinoma remains debated. In appropriately selected patients, current thyroid cancer management has increasingly moved toward a more conservative operative approach. Thyroid lobectomy may be preferred over total thyroidectomy when oncologically suitable, as it can limit exposure to complications such as recurrent laryngeal nerve injury, permanent hypoparathyroidism, and the need for lifelong thyroid hormone replacement without compromising oncologic outcomes [1-3].

A critical rationale for performing total thyroidectomy is the reported 10% to 30% incidence of occult contralateral carcinoma, referring to malignant disease in the thyroid lobe opposite the index tumor that is not recognized on preoperative imaging and is only confirmed after final histopathologic examination [4,5]. However, subjecting all patients with clinically solitary papillary thyroid carcinoma to total thyroidectomy for this possibility may constitute overtreatment for the majority who do not harbor contralateral disease. This issue is particularly relevant in the Philippines, where studies have identified Filipino cohorts as having a high incidence of thyroid cancer and, in some reports, more aggressive clinicopathologic features [6-9]. Surgical decision-making is further complicated by the coexistence of international and local guidelines, which may contribute to variation in practice [1,10-13].

To address this clinical dilemma, Feng et al. developed a practical 10-point risk-scoring model for contralateral occult carcinoma based on five variables: tumor size greater than 1 cm, presence of a benign nodule, extrathyroidal extension, central lymph node metastasis, and lateral lymph node metastasis [4]. A score of four or higher was associated with an increased risk of occult contralateral carcinoma and demonstrated strong diagnostic performance in the original cohort [4]. However, although “presence of benign nodule” was included in the model, Feng et al. did not provide a detailed operational definition based on TI-RADS, specific ultrasound features, cytology, or histopathology. Their cohort consisted of patients with a single papillary thyroid carcinoma confined to one lobe and no suspicious carcinoma lesions in the contralateral lobe on preoperative imaging or fine-needle aspiration [4].

Zhang et al. also reported that tumor size greater than 1 cm, ipsilateral multifocality, contralateral benign nodules, and central lymph node metastasis were associated with contralateral occult papillary thyroid carcinoma [5]. Their study similarly excluded patients with suspicious contralateral lesions on preoperative assessment and did not provide a detailed TI-RADS-based definition of benign nodules. Thus, “contralateral benign nodules” should be understood as contralateral nodules not considered suspicious or malignant before surgery, rather than nodules necessarily proven benign by separate biopsy or histopathology [5]. By comparison, extrathyroidal extension and lateral lymph node metastasis showed less consistent associations with contralateral occult disease [5].

Although the Feng model provides a practical risk-stratification framework, its direct use for preoperative surgical decision-making is limited. The variables in the original model were based on postoperative clinicopathologic findings obtained after total thyroidectomy, which are not available when making the initial choice between thyroid lobectomy and total thyroidectomy [4]. While this supports the prediction of occult contralateral carcinoma after complete pathologic evaluation, it limits the model’s direct usefulness for deciding the initial extent of surgery. In addition, its applicability outside the original Chinese cohort remains uncertain. The present study evaluated an exploratory preoperative adaptation of the Feng-derived score using information available before surgery, including preoperative ultrasonography and physical examination findings. This modification was intended to assess whether the score could provide preoperative risk information using data available before the initial surgical decision.

This study aimed to evaluate the apparent diagnostic utility of a modified preoperative adaptation of the Feng-derived risk score for predicting occult contralateral carcinoma among Filipino patients with clinically solitary papillary thyroid carcinoma, using preoperative clinical and ultrasonographic findings to assign the score, with final histopathology after total thyroidectomy as the reference standard.

## Materials and methods

Study design and setting

We conducted a retrospective diagnostic accuracy study at Chong Hua Hospital, Cebu, a tertiary care institution in Cebu City, Philippines. The study examined how well a preoperative adaptation of the Feng-derived risk score identified occult contralateral carcinoma in adults with clinically solitary papillary thyroid carcinoma. The modified risk score served as the index test, while the presence or absence of contralateral carcinoma on final histopathologic examination after total thyroidectomy served as the reference standard. Reporting was guided by the 2015 Standards for Reporting Diagnostic Accuracy Studies recommendations [14].

Study population

The cohort consisted of patients at least 18 years old who underwent total thyroidectomy for clinically solitary papillary thyroid carcinoma from January 1, 2019, to December 31, 2023, with or without central or lateral neck dissection. Patients were considered eligible if papillary thyroid carcinoma had been diagnosed preoperatively by fine-needle aspiration biopsy classified as Bethesda V or VI, and if complete records were available, including physical examination findings, preoperative neck ultrasound reports, operative documentation, and final histopathology results.

Patients were excluded if they had preoperative ultrasonographic evidence of bilateral suspicious thyroid lesions, contralateral malignant-appearing nodules, or clinically evident contralateral carcinoma. Patients were also excluded if they had previous thyroid surgery, final histopathology not corresponding to papillary thyroid carcinoma, age younger than 18 years, or incomplete medical records. All patients who met the eligibility criteria during the defined study period were included in the analysis. The final analytic cohort consisted of 135 patients, including 19 patients with occult contralateral carcinoma and 116 without occult contralateral carcinoma. Because the number of outcome events was limited, the study was interpreted as an exploratory assessment of diagnostic utility rather than as definitive external validation or redevelopment of the original Feng model.

Data collection and variables

Study data were obtained from hospital medical records and recorded using a uniform data extraction form. The variables reviewed included patient age, sex, preoperative tumor size, benign nodule status, suspected extrathyroidal extension, suspected central and lateral lymph node metastases, fine-needle aspiration biopsy category, type of operation performed, and final histopathologic findings.

For the modified preoperative score, all variables were based solely on information available before surgery, including physical examination and preoperative neck ultrasonography. Benign nodules were defined as additional thyroid nodules described in the preoperative ultrasound report as benign-appearing, probably benign, low-suspicion, non-suspicious, or without suspicious malignant features. This classification was based on the original preoperative ultrasound interpretation and did not require separate fine-needle aspiration biopsy, benign cytology, or histopathologic confirmation. When TI-RADS classification was available, low-suspicion or non-suspicious nodules were considered benign-appearing for purposes of the score. However, TI-RADS classification was not required because the study relied on retrospective review of routine preoperative ultrasound reports, and TI-RADS scoring was not uniformly documented across all cases. Suspicious or malignant-appearing contralateral nodules were excluded because these represented clinically detectable contralateral disease rather than occult contralateral carcinoma.

Suspected extrathyroidal extension was defined as ultrasound-reported concern for tumor extension beyond the thyroid capsule, capsular disruption, loss of the normal thyroid boundary, or involvement or abutment of adjacent soft tissue or strap muscles. Suspected central lymph node metastasis refers to abnormal or suspicious lymph nodes reported in the central neck compartment, including level VI and/or the prelaryngeal, pretracheal, and paratracheal nodal basins. Suspected lateral lymph node metastasis refers to abnormal or suspicious lymph nodes identified in the lateral neck compartments, including levels II-V.

Lymph nodes were considered suspicious when the ultrasound report described features suggestive of metastatic involvement, including pathologic enlargement, particularly increased short-axis diameter when accompanied by suspicious morphology; loss of the fatty hilum; rounded shape; cystic change; microcalcifications or punctate echogenic foci; hyperechogenicity; peripheral or abnormal vascularity; or irregular margins. Size criteria, such as short-axis diameter greater than 8-10 mm, were considered when reported; however, lymph node classification was ultimately based on the interpreting physician’s preoperative ultrasound impression. Because this was a retrospective study that relied on routine clinical ultrasound reports rather than a prospectively standardized imaging protocol, these variables were not uniformly assessed or documented across all cases.

Index test

The preoperative risk score was assigned retrospectively, using only variables documented before surgery. The scoring system was modified from the 10-point model described by Feng et al. and was based on findings from preoperative neck ultrasound and physical examination. One point was given for a tumor diameter greater than 1 cm; two points for the presence of benign-appearing nodules; three points for suspected extrathyroidal extension; two points for suspected central compartment lymph node metastasis; and two points for suspected lateral neck lymph node metastasis [4]. The total score ranged from zero to 10. A cutoff score of four or higher was used to classify patients as high risk, corresponding to the threshold identified by Youden’s index in this cohort and consistent with the cutoff proposed in the original Feng model [4]. To reduce review bias, score assignment was performed before the extraction and classification of the final histopathology outcome. Thus, the investigators assigning the score were blinded to the presence or absence of occult contralateral carcinoma at the time the index test was determined.

Reference standard and outcome definition

Occult contralateral carcinoma was defined as papillary thyroid carcinoma identified in the contralateral thyroid lobe on final histopathology that had not been detected as malignant disease on preoperative ultrasonography. After score assignment, final histopathology reports were reviewed to classify patients as occult contralateral carcinoma-positive or occult contralateral carcinoma-negative. Because histologic confirmation of occult contralateral carcinoma requires removal of the contralateral thyroid lobe, the study population was limited to patients who underwent total thyroidectomy. Therefore, the reference standard could not be applied to patients treated with thyroid lobectomy alone, creating verification bias.

The index-test variables were derived from preoperative records, while the reference-standard outcome was derived from postoperative final histopathology. To reduce incorporation bias, the preoperative score was calculated using only information available before surgery, while the outcome was defined only by the final histopathology report. Patients with incomplete records were excluded, and no imputation was performed.

Statistical analysis

Statistical analysis was performed using SPSS Statistics version 26 (SPSS Statistics version 26.0 (IBM Corp. Released 2019. IBM SPSS Statistics for Windows, Version 26.0. Armonk, NY: IBM Corp.). Patient characteristics were summarized descriptively. Categorical data were reported as counts and percentages, whereas continuous data were expressed as means with standard deviations. The cohort was grouped by the presence or absence of occult contralateral carcinoma on final histopathologic examination. Comparisons between groups were performed using the chi-square test or Fisher’s exact test for categorical variables, as appropriate, and the independent samples t-test for continuous variables.

Diagnostic accuracy was determined from two-by-two contingency tables. Performance measures included sensitivity, specificity, positive and negative predictive values, positive and negative likelihood ratios, and overall accuracy, each reported with corresponding 95% CIs. CIs for sensitivity, specificity, predictive values, disease prevalence, and accuracy were calculated using binomial methods, while CIs for likelihood ratios were calculated using standard log-transformed methods. Discrimination was assessed using receiver operating characteristic curve analysis, with the area under the curve and its 95% CI reported. The cutoff with the best combined sensitivity and specificity was selected using Youden’s index, defined as the sum of sensitivity and specificity minus 1. Because this cutoff was derived and evaluated in the same cohort, the reported threshold and associated diagnostic estimates should be interpreted as apparent performance. They may overestimate performance in an independent cohort. Because predictive values vary with disease prevalence, they were interpreted relative to the observed prevalence in the study cohort.

Ethical considerations

The protocol received approval from the Chong Hua Hospital Institutional Review Board (approval number: IRBi-2025-108) before the start of data collection. To protect confidentiality, all personal identifiers were removed and replaced with assigned study codes. The collected data were kept secure and used only for this research. Given the retrospective design and the use of de-identified records, informed consent was waived in accordance with the ethics approval. The investigators reported no conflicts of interest.

## Results

Patient characteristics

A total of 135 adult patients with clinically solitary papillary thyroid carcinoma who underwent total thyroidectomy met the inclusion criteria and were included in the final analysis. Occult contralateral carcinoma was identified on final histopathology in 19 patients, corresponding to a prevalence of 14.1%. The remaining 116 patients had no occult contralateral carcinoma on final histopathology.

The mean age of the overall cohort was 39.59 ± 13.26 years. Patients with occult contralateral carcinoma had a mean age of 42.05 ± 12.74 years, while patients without occult contralateral carcinoma had a mean age of 39.11 years. There was no statistically significant difference in age between patients with and without occult contralateral carcinoma. The cohort included 35 male patients and 100 female patients. Sex was not significantly associated with occult contralateral carcinoma.

The demographic characteristics of the study population are summarized in Table 1.

**Table 1:** Demographic profile of the study population Categorical values are presented as n (%). OCC: occult contralateral carcinoma, SD: standard deviation

Characteristic	All patients (N = 135)	OCC-positive (n = 19)	OCC-negative (n = 116)	p-value
Age, years, mean ± SD	39.59 ± 13.26	42.05 ± 12.74	39.11 ± 13.35	0.356
Male sex	35 (25.9%)	7 (36.8%)	28 (24.1%)	0.264
Female sex	100 (74.1%)	12 (63.2%)	88 (75.9%)	0.264

Clinicoradiologic characteristics

Most patients had tumors greater than 1 cm, with 115 patients classified in this category and 20 patients having tumors measuring 1 cm or less. Tumor size greater than 1 cm was not significantly associated with occult contralateral carcinoma.

Benign nodules were present in 16 patients and absent in 119 patients. The presence of benign nodules was significantly associated with occult contralateral carcinoma. Among patients with occult contralateral carcinoma, 10 of 19 had benign nodules, compared with six of 116 patients without occult contralateral carcinoma.

Suspected extrathyroidal extension was identified in three patients. No significant association was observed between suspected extrathyroidal extension and occult contralateral carcinoma. Suspected central lymph node metastasis was present in 12 patients and was significantly associated with occult contralateral carcinoma. Suspected lateral lymph node metastasis was present in 20 patients and was also significantly associated with occult contralateral carcinoma.

The clinicoradiologic characteristics according to occult contralateral carcinoma status are presented in Table 2.

**Table 2:** Clinicoradiologic characteristics according to occult contralateral carcinoma status Values are presented as n (%). OCC: occult contralateral carcinoma

Characteristic	Category	All patients (N = 135)	OCC-positive (n = 19)	OCC-negative (n = 116)	p-value
Tumor size	≤ 1 cm	20 (14.8%)	1 (5.3%)	19 (16.4%)	0.306
	>1 cm	115 (85.2%)	18 (94.7%)	97 (83.6%)	
Benign nodule	Absent	119 (88.1%)	9 (47.4%)	110 (94.8%)	<0.001
	Present	16 (11.9%)	10 (52.6%)	6 (5.2%)	
Suspected extrathyroidal extension	Absent	132 (97.8%)	19 (100.0%)	113 (97.4%)	1.000
	Present	3 (2.2%)	0 (0.0%)	3 (2.6%)	
Suspected central lymph node metastasis	Absent	123 (91.1%)	14 (73.7%)	109 (94.0%)	0.014
	Present	12 (8.9%)	5 (26.3%)	7 (6.0%)	
Suspected lateral lymph node metastasis	Absent	115 (85.2%)	11 (57.9%)	104 (89.7%)	0.002
	Present	20 (14.8%)	8 (42.1%)	12 (10.3%)	

Receiver operating characteristic curve analysis

A receiver operating characteristic curve was generated to assess the ability of the modified preoperative Feng-derived score to distinguish patients with occult contralateral carcinoma from those without. The score achieved an area under the curve of 0.910, with a standard error of 0.033 and a 95% CI ranging from 0.845 to 0.976, indicating strong apparent discrimination within this cohort. However, only 19 patients were occult contralateral carcinoma-positive, and no internal validation, such as bootstrapping or cross-validation, was performed; therefore, this estimate may be unstable and optimistic.

Receiver operating characteristic curve analysis results are shown in Table 3.

**Table 3:** Area under the receiver operating characteristic curve CI: confidence interval

Area under the curve	Standard error	p-value	95% CI lower bound	95% CI upper bound
0.910	0.033	<0.001	0.845	0.976

**Figure 1:**
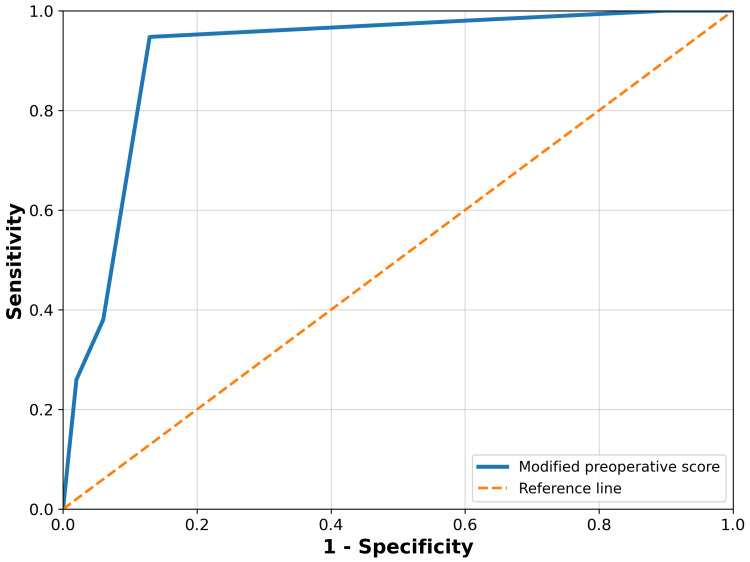
Receiver operating characteristic curve of the modified preoperative risk score The receiver operating characteristic curve shows the discriminatory performance of the modified preoperative Feng-derived risk score for predicting occult contralateral carcinoma. The area under the curve was 0.910 with a 95% CI of 0.845-0.976. CI: confidence interval

The receiver operating characteristic curve for the modified preoperative risk score is shown in Figure 1.

Diagnostic performance of the modified preoperative score

The Youden-derived optimal threshold in this cohort was 3.5, corresponding clinically to a score of four or higher. This threshold was the same as the cutoff proposed in the original Feng model; however, in the present study, it was selected and evaluated in the same dataset. Therefore, the cutoff should be interpreted as exploratory and not externally validated. At this threshold, the modified preoperative score showed an apparent sensitivity of 94.7% and an apparent specificity of 87.1%.

At this threshold, the score achieved a positive predictive value of 54.6%, a negative predictive value of 99.0%, and an overall accuracy of 88.2%. The corresponding positive and negative likelihood ratios were 7.33 and 0.06, respectively. The two-by-two classification table showed 18 true positives, one false negative, 101 true negatives, and 15 false positives. Although the score showed high sensitivity and negative predictive value, its positive predictive value was modest. Thus, a high-risk score should not be used to confirm occult contralateral carcinoma or to independently justify total thyroidectomy; instead, it should be interpreted together with guideline-based risk stratification and patient-specific clinical factors.

The diagnostic performance of the modified preoperative Feng-derived risk score at the selected cutoff is summarized in Table 4.

**Table 4:** Diagnostic performance of the modified preoperative Feng-derived risk score Predictive values and disease prevalence are prevalence-dependent. OCC: occult contralateral carcinoma, CI: confidence interval

Statistic	Value	95% CI
Threshold using the Youden index	3.5	Not applicable
Sensitivity	94.74%	74.0-99.9
Specificity	87.07%	79.6-92.6
Positive likelihood ratio	7.33	4.61-11.65
Negative likelihood ratio	0.06	0.01-0.40
Disease prevalence	14.07%	9.20-20.94
Positive predictive value	54.55%	37.99-70.16
Negative predictive value	99.02%	94.66-99.83
Accuracy	88.15%	81.61-92.57
True positive/false negative	18/1	Based on OCC-positive patients (n = 19)
True negative/false positive	101/15	Based on OCC-negative patients (n = 116)

## Discussion

Principal finding

This study evaluated the exploratory diagnostic utility of a modified preoperative Feng-derived risk score for predicting occult contralateral carcinoma among Filipino patients with clinically solitary papillary thyroid carcinoma. In this cohort of 135 patients who underwent total thyroidectomy, occult contralateral carcinoma was identified in 19 patients, corresponding to a prevalence of 14.1%. The modified score demonstrated strong apparent discrimination, with an area under the curve of 0.910. At a cutoff score of four or higher, the model showed a sensitivity of 94.7%, specificity of 87.1%, negative predictive value of 99.0%, and overall accuracy of 88.2%.

The high negative predictive value is the most clinically relevant finding. In practical terms, a low score may help identify patients with a lower likelihood of occult contralateral carcinoma. However, the positive predictive value was only 54.6%, meaning that a high score did not reliably confirm the presence of occult contralateral disease. Therefore, the modified score appears more suitable as a potential preoperative rule-out adjunct than as a rule-in tool. This distinction is important because a high-risk score should not automatically justify total thyroidectomy. The decision between thyroid lobectomy and total thyroidectomy should remain based on established guideline recommendations, tumor characteristics, nodal status, patient preference, surgeon judgment, and institutional practice [1,10-13].

Comparison with previous studies

The original Feng model was developed to estimate the risk of occult contralateral carcinoma in patients with clinically solitary papillary thyroid carcinoma using a 10-point scoring system [4]. A score of four or higher was associated with increased risk of occult contralateral carcinoma and demonstrated strong diagnostic performance in the original cohort [4]. However, several variables in the original Feng model were based on postoperative clinicopathologic findings obtained after total thyroidectomy, including histopathologic assessment of extrathyroidal extension and lymph node metastasis [4]. This limits the model’s direct usefulness for deciding the initial extent of surgery, because the choice between thyroid lobectomy and total thyroidectomy must be made before final histopathologic information is available.

The present study adapted the Feng model by using preoperative ultrasonographic and physical examination findings instead of postoperative histopathologic variables. This distinction is central to the clinical purpose of the study. The modified score is not a purely external validation of the original clinicopathologic model; rather, it is an exploratory assessment of the diagnostic utility of a preoperative adaptation. Despite this modification, the area under the curve in this cohort was similar to the diagnostic performance reported in the original Feng study [4]. This suggests that the general scoring concept may have preliminary discriminatory value when adapted to preoperative information, although this requires confirmation in independent cohorts.

The prevalence of occult contralateral carcinoma in this study was 14.1%, which falls within the 10% to 30% range reported in previous literature [4,5]. In the study by Zhang et al., factors linked to occult disease in the opposite lobe included tumor diameter greater than 1 cm, multifocal tumors, benign nodules in the contralateral lobe, and central compartment lymph node metastasis. Their findings for extrathyroidal extension and lateral neck lymph node metastasis were less uniform [5]. In our analysis, the presence of benign nodules and suspected nodal involvement in either the central or lateral neck compartments showed significant associations with occult contralateral carcinoma, whereas tumor size greater than 1 cm and suspected extrathyroidal extension did not.

Interpretation of individual predictors

Tumor size greater than 1 cm was not significantly associated with occult contralateral carcinoma in this cohort. This may be explained by limited variation in tumor size, since most patients in the study had tumors greater than 1 cm. When one category contains most of the cohort, the ability to detect a meaningful association is reduced. Therefore, this finding should not be interpreted as definitive evidence that tumor size is unrelated to contralateral disease.

The presence of benign nodules was strongly associated with occult contralateral carcinoma. This supports careful assessment of the entire thyroid gland during preoperative ultrasonography, even when the index lesion appears clinically solitary. In this study, benign nodules were defined as additional thyroid nodules described on preoperative ultrasonography as benign-appearing or non-suspicious for malignancy. Suspicious or malignant-appearing contralateral nodules were excluded because these would represent clinically detectable contralateral disease rather than occult disease.

Suspected extrathyroidal extension was uncommon in this cohort, with only three patients classified as having this finding preoperatively. No significant association was observed between suspected extrathyroidal extension and occult contralateral carcinoma. The small number of patients with suspected extrathyroidal extension likely limits the generalizability of this result. It should not be interpreted as evidence that extrathyroidal extension is clinically unimportant in papillary thyroid carcinoma.

Both suspected central and lateral lymph node metastases were significantly associated with occult contralateral carcinoma. This supports the concept that nodal disease may serve as a marker of more diffuse tumor behavior. The association with central lymph node metastasis is consistent with previous evidence identifying it as a risk factor for occult contralateral carcinoma [5]. The association with lateral lymph node metastasis should be interpreted cautiously because the number of patients with suspected lateral nodal disease was limited, and previous studies have reported less consistent findings for this variable [5].

Clinical implications

The findings of this study are clinically relevant because the optimal extent of surgery for selected patients with clinically unilateral papillary thyroid carcinoma remains debated. Total thyroidectomy may address the possibility of occult contralateral carcinoma and facilitate postoperative surveillance, but it carries a greater risk of complications and lifelong hormone replacement compared with thyroid lobectomy [1-3]. Conversely, thyroid lobectomy may reduce treatment-related morbidity in appropriately selected patients but may leave occult contralateral disease untreated.

In this context, a preoperative score with a high negative predictive value may provide adjunctive information during shared decision-making. A low score may support the discussion that the likelihood of occult contralateral carcinoma is low. However, because the positive predictive value was modest and false-positive results occurred, a high score should not be interpreted as confirming occult contralateral disease or as an automatic indication for total thyroidectomy. Instead, a high score should prompt individualized counseling informed by established guideline recommendations, tumor characteristics, nodal assessment, patient-specific risk factors, surgeon judgment, institutional practice, and patient preferences [1,10-13]. Importantly, because this study included only patients who underwent total thyroidectomy, the findings should be applied cautiously to patients who are otherwise appropriate candidates for lobectomy.

This issue is especially relevant in the Philippine setting. Previous studies have reported a high incidence of thyroid cancer among Filipino cohorts and, in some cohorts, more aggressive clinicopathologic features [6-9]. Surgical decision-making may also be influenced by differences between international and local recommendations regarding the extent of surgery for papillary thyroid carcinoma [1,10-13]. A locally evaluated preoperative risk score may help provide a more individualized framework for counseling Filipino patients, but further validation is necessary before it can be incorporated into routine clinical practice.

Strengths and limitations

A major strength of this study is its clinically relevant design. The score was calculated using information available before surgery, making it more applicable to the actual decision between thyroid lobectomy and total thyroidectomy. The study also used final histopathology after total thyroidectomy as the reference standard, which allowed direct confirmation of occult contralateral carcinoma.

Several limitations should be considered when interpreting these findings. The study used a retrospective design and was conducted at a single tertiary hospital, which may limit the generalizability of the results to other settings. The area under the curve and other diagnostic performance estimates may be unstable, imprecise, and optimistic because only 19 patients were occult contralateral carcinoma-positive, and no internal validation, such as bootstrapping or cross-validation, was performed. The cutoff was selected using Youden’s index in the same dataset used to estimate diagnostic performance; therefore, the threshold should not be considered externally validated, and the associated accuracy estimates may be optimistic.

Because histologic confirmation of occult contralateral carcinoma requires removal of the contralateral thyroid lobe, this study was limited to patients who underwent total thyroidectomy. This introduces verification bias, since patients treated with lobectomy alone could not have the reference standard applied to the contralateral lobe. It also introduces potential selection bias because patients selected for total thyroidectomy may already have had higher-risk clinical features, surgeon- or institution-specific indications, or patient-related factors that differed from those of patients selected for lobectomy. Patients who were truly eligible for lobectomy may therefore be underrepresented in this cohort. As a result, the calculated sensitivity, specificity, predictive values, likelihood ratios, and overall accuracy should be interpreted as apparent diagnostic performance within a total-thyroidectomy cohort rather than as estimates directly generalizable to all patients with clinically solitary papillary thyroid carcinoma. These estimates may differ in a broader population that includes lobectomy-treated or lobectomy-eligible patients, who may have lower-risk clinical profiles and a different prevalence of occult contralateral carcinoma. Because predictive values are prevalence-dependent, the positive and negative predictive values observed in this study may not apply directly to patients being considered for lobectomy. This limits the applicability of the findings to the population in whom the choice between thyroid lobectomy and total thyroidectomy is most clinically relevant.

Another limitation is that preoperative ultrasound findings were taken from routine clinical reports rather than from a prospectively standardized imaging protocol. TI-RADS classification and detailed nodal measurements were not uniformly documented across all cases; therefore, classification of benign-appearing nodules, suspected extrathyroidal extension, and suspected central or lateral lymph node metastasis depended on the original interpreting physician’s report. This may have introduced variability in how the ultrasound-based score variables were assessed. Although score assignment was performed before extraction and classification of final histopathology outcomes, the retrospective use of routine clinical records may still have introduced abstraction bias. Finally, this study evaluated a preoperative modification of the Feng scoring system rather than directly validating the original clinicopathologic model.

Future directions

Future studies should prospectively validate this modified preoperative score in larger, multicenter Filipino cohorts using standardized ultrasonographic definitions for benign nodules, extrathyroidal extension, and central and lateral lymph node metastases. Larger sample sizes with more occult contralateral carcinoma events would allow more precise estimates of sensitivity, specificity, calibration, and clinical utility. Future research should also evaluate long-term outcomes among low-score patients managed with thyroid lobectomy to determine whether the score can contribute to surgical de-escalation discussions in selected patients.

## Conclusions

This exploratory single-center retrospective diagnostic accuracy study found that a modified Feng-derived preoperative risk score demonstrated strong apparent discrimination for occult contralateral carcinoma among Filipino patients with clinically solitary papillary thyroid carcinoma who underwent total thyroidectomy. Its high negative predictive value suggests that the score may be most useful as a potential preoperative rule-out adjunct during counseling, particularly when interpreted alongside established guideline-based risk stratification, tumor characteristics, nodal assessment, patient preferences, and surgeon judgment.

However, the score should not be used as a standalone surgical decision rule or as an independent determinant of surgical extent. Its modest positive predictive value limits its utility for confirming occult contralateral carcinoma, and a high score should not be interpreted as confirming occult contralateral carcinoma or automatically justifying total thyroidectomy. Because this study was retrospective, conducted at a single center, included a limited number of occult contralateral carcinoma events, selected and tested the cutoff in the same cohort, and evaluated only patients who underwent total thyroidectomy, the findings should be interpreted as apparent diagnostic performance. Prospective multicenter validation with standardized ultrasound definitions and long-term oncologic follow-up is needed before the score can be incorporated into routine surgical decision-making.
